# Supplementary material for: The Association between Prenatal Exposure to Per- and Polyfluoroalkyl Substances and Respiratory Tract Infections in Preschool Children: A Wuhan Cohort Study
Source: Toxics. 2023 Nov 2;11(11):897. doi: 10.3390/toxics11110897 (PMC10674762; doi:10.3390/toxics11110897)
Supplement: Supplementary file 1 [file toxics-11-00897-s001.zip › toxics-2675854-supplementary.pdf]

Table S1. Names, chemical formulas, and molecular structures of target compounds.

**Abbreviation**	**Full name**	**CAS number**	**Chemical formula**
PFOA	perfluorooctanoic acid	335-67-1	$C_8HF_{15}O_2$
PFNA	perfluorononanoic acid	375-95-1	$C_9HF_{17}O_2$
PFDA	perfluorodecanoic acid	335-76-2	$C_{10}HF_{19}O_2$
PFUnDA	perfluoroundecanoic acid	2058-94-8	$C_{11}HF_{21}O_2$
PFDoDA	perfluorododecanoic acid	307-55-1	$C_{12}HF_{23}O_2$
PFTrDA	perfluorotridecanoic acid	72629-94-8	$C_{13}HF_{25}O_2$
PFHxS	perfluorohexane sulfonic acid	355-46-4	$C_6HF_{13}O_3S$
PFOS	perfluorooctane sulfonic acid	1763-23-1	$C_8HF_{17}O_3S$
6:2 Cl-PFESA	6:2 chlorinated polyfluorinated ether sulfonate	73606-19-6	$C_8ClF_{16}KO_4S$
8:2 Cl-PFESA	8:2 chlorinated polyfluorinated ether sulfonate	83329-89-9	$C_{10}ClF_{20}KO_4S$

Table S2. General demographic characteristics of pregnant women included in the study.

Characteristics	Mean ± SD or n (%)
Maternal age at delivery, years	29.12 ± 3.14
< 25	29 (5.50)
25 ~ 29	263 (49.91)
≥30	235 (44.59)
Pre-pregnancy BMI, kg/m^2	20.64 ± 2.48
Underweight (< 18.5)	93 (17.65)
Normal (18.5 ~ 23.9)	380 (72.11)
Overweight or obesity (> 24)	54 (10.10)
Education status, years	
< 13	85 (16.13)
13	399 (75.71)
> 13	43 (8.16)
Annual household income, ten thousand yuan	
< 10	331 (62.81)
≥ 10	194 (36.81)
Null	2 (0.38)
Passive smoking during pregnancy (women)	
Yes	143 (27.13)
No	384 (72.87)
Parity	
Primiparous	478 (90.70)
Multiparous	49 (9.30)
Neonatal sex	
Boys	284 (53.89)
Girls	243 (46.11)
Gestational age at birth, weeks	39.32 ± 1.12
Preterm birth (< 37)	17 (3.23)
Term birth (37 ~ 42)	510 (96.77)
Birth weight, kg	3.34 ± 0.41
Low birth weight (< 2.5)	9 (1.71)
Normal birth weight (2.5 ~ 4.0)	490 (92.98)
Macrosomia (> 4.0)	28 (5.31)
Breastfeeding months, months	
< 6	142 (26.94)
≥ 6	347 (65.84)
Null	38 (7.21)
Passive smoking during pregnancy (children)	
Yes	252 (47.82)
No	262 (49.72)
Null	13 (2.47)

Abbreviation: SD, standard deviation; BMI, body mass index.

Table S3. Association between single PFASs and RTIs after excluding preterm birth, low birth weight, and macrosomia.

	Incidence [a]		Frequency [b]	
	OR (95% CI)	*P*	β (95% CI)	*P*
PFOA (ng/mL)				
Common cold	1.00 (0.65,1.54)	0.998	-0.05 (-0.18,0.07)	0.394
Bronchitis	1.15 (0.79,1.66)	0.469	0.12 (-0.09,0.33)	0.272
Pneumonia	0.95 (0.55,1.64)	0.864	-0.24 (-0.73,0.24)	0.330
Tonsillitis	1.07 (0.69,1.67)	0.756	-0.03 (-0.32,0.27)	0.859
PFNA (ng/mL)				
Common cold	0.79 (0.55,1.13)	0.201	-0.04 (-0.14,0.06)	0.448
Bronchitis	1.07 (0.79,1.45)	0.650	0.01 (-0.17,0.19)	0.924
Pneumonia	0.98 (0.63,1.52)	0.924	-0.16 (-0.54,0.21)	0.388
Tonsillitis	1.25 (0.87,1.80)	0.226	0.20 (-0.05,0.44)	0.115
PFDA (ng/mL)				
Common cold	0.84 (0.63,1.13)	0.254	0.02 (-0.06,0.11)	0.582
Bronchitis	1.00 (0.77,1.28)	0.970	-0.03 (-0.18,0.11)	0.660
Pneumonia	1.01 (0.70,1.46)	0.967	-0.16 (-0.48,0.16)	0.314
Tonsillitis	1.25 (0.92,1.69)	0.150	**0.20 (0.00,0.40)**	**0.046***
PFUnDA (ng/mL)				
Common cold	0.88 (0.64,1.21)	0.432	0.06 (-0.03,0.15)	0.194
Bronchitis	1.00 (0.77,1.31)	0.976	-0.02 (-0.18,0.14)	0.800
Pneumonia	1.07 (0.72,1.59)	0.730	-0.07 (-0.40,0.26)	0.684
Tonsillitis	1.30 (0.94,1.79)	0.120	**0.22 (0.00,0.44)**	**0.046***
PFDoDA (ng/mL)				
Common cold	0.99 (0.76,1.27)	0.914	0.07 (0.00,0.15)	0.052
Bronchitis	1.01 (0.81,1.26)	0.936	0.00 (-0.12,0.12)	0.982
Pneumonia	1.08 (0.78,1.49)	0.651	0.04 (-0.25,0.33)	0.811
Tonsillitis	1.31 (1.00,1.72)	0.051	**0.29 (0.11,0.47)**	**0.001***
PFTrDA (ng/mL)				
Common cold	0.99 (0.73,1.35)	0.959	0.08 (-0.01,0.18)	0.078
Bronchitis	1.05 (0.80,1.38)	0.728	0.01 (-0.15,0.17)	0.889
Pneumonia	1.05 (0.70,1.57)	0.807	-0.03 (-0.37,0.32)	0.868
Tonsillitis	1.29 (0.93,1.78)	0.128	0.20 (-0.02,0.42)	0.071
PFHxS (ng/mL)				
Common cold	1.11 (0.80,1.53)	0.539	-0.04 (-0.13,0.06)	0.442
Bronchitis	1.04 (0.79,1.37)	0.773	-0.09 (-0.25,0.07)	0.261
Pneumonia	1.26 (0.84,1.87)	0.260	0.29 (-0.05,0.63)	0.099
Tonsillitis	1.09 (0.79,1.51)	0.601	0.01 (-0.21,0.22)	0.962
PFOS (ng/mL)				
Common cold	0.95 (0.76,1.19)	0.661	-0.01 (-0.07,0.05)	0.768
Bronchitis	0.97 (0.80,1.18)	0.772	-0.09 (-0.20,0.02)	0.106
Pneumonia	1.08 (0.82,1.42)	0.591	-0.02 (-0.27,0.23)	0.887
Tonsillitis	0.98 (0.78,1.24)	0.891	-0.02 (-0.17,0.13)	0.792

	Incidence [a]		Frequency [b]	
	OR (95% CI)	*P*	β (95% CI)	*P*
6:2 Cl-PFESA (ng/mL)				
Common cold	0.98 (0.76,1.25)	0.861	0.01 (-0.06,0.08)	0.765
Bronchitis	1.06 (0.85,1.31)	0.602	0.05 (-0.08,0.17)	0.457
Pneumonia	1.06 (0.78,1.45)	0.711	-0.12 (-0.39,0.15)	0.380
Tonsillitis	1.16 (0.90,1.50)	0.243	0.14 (-0.03,0.31)	0.098
8:2 Cl-PFESA (ng/mL)				
Common cold	0.92 (0.74,1.14)	0.442	0.01 (-0.06,0.07)	0.834
Bronchitis	1.06 (0.88,1.28)	0.542	0.01 (-0.10,0.11)	0.916
Pneumonia	1.04 (0.79,1.36)	0.806	-0.15 (-0.41,0.11)	0.250
Tonsillitis	1.14 (0.91,1.43)	0.250	**0.19 (0.05,0.33)**	**0.008***

Abbreviation: OR, odds ratio; CI, confidence interval. The model was adjusted for maternal age (continuous variable), family annual income (< 100,000 yuan; ≥ 100,000 yuan), child sex (male; female), gestational week (continuous variable), parity (primipara; multipara), breastfeeding duration (< 6 months; ≥6 months), and child exposure to passive smoking (yes; no). These potential confounders were adjusted for in the regression model. *: $P<0.05$. [a] Logistic regression. [b] Poisson regression.

Table S4. Association between single PFASs and the incidence of RTIs in the study population stratified by sex.

	Boys		Girls		$P_{interaction}$
	OR (95% CI)	*P*	OR (95% CI)	*P*	
PFOA (ng/mL)					
Common cold	0.80 (0.48,1.34)	0.405	1.72 (0.92,3.19)	0.087	0.078
Bronchitis	1.04 (0.67,1.61)	0.870	1.10 (0.64,1.90)	0.725	0.763
Pneumonia	1.22 (0.64,2.33)	0.543	0.51 (0.23,1.15)	0.105	0.087
Tonsillitis	0.80 (0.46,1.37)	0.414	1.74 (0.89,3.38)	0.104	0.083
PFNA (ng/mL)					
Common cold	0.71 (0.44,1.13)	0.145	1.00 (0.59,1.71)	0.995	0.307
Bronchitis	1.00 (0.69,1.46)	0.993	1.18 (0.74,1.90)	0.482	0.619
Pneumonia	1.03 (0.59,1.79)	0.916	0.75 (0.38,1.48)	0.406	0.381
Tonsillitis	1.26 (0.81,1.98)	0.306	1.48 (0.84,2.59)	0.176	0.461
PFDA (ng/mL)					
Common cold	0.76 (0.52,1.13)	0.175	1.01 (0.65,1.56)	0.969	0.322
Bronchitis	0.99 (0.72,1.36)	0.952	1.08 (0.73,1.59)	0.712	0.916
Pneumonia	1.08 (0.67,1.74)	0.750	0.80 (0.46,1.40)	0.439	0.316
Tonsillitis	1.34 (0.91,1.99)	0.138	1.25 (0.78,1.99)	0.357	0.979
PFUnDA (ng/mL)					
Common cold	0.83 (0.55,1.25)	0.374	1.03 (0.64,1.65)	0.904	0.447
Bronchitis	0.98 (0.70,1.38)	0.909	1.17 (0.77,1.78)	0.466	0.697
Pneumonia	1.15 (0.69,1.94)	0.585	0.85 (0.46,1.54)	0.589	0.366
Tonsillitis	1.21 (0.81,1.80)	0.361	1.49 (0.89,2.47)	0.127	0.454
PFDoDA (ng/mL)					
Common cold	1.03 (0.73,1.44)	0.874	1.03 (0.72,1.47)	0.870	0.948
Bronchitis	1.07 (0.80,1.44)	0.653	1.00 (0.73,1.37)	0.991	0.566
Pneumonia	1.26 (0.80,1.98)	0.325	0.85 (0.54,1.32)	0.463	0.203
Tonsillitis	1.32 (0.93,1.90)	0.124	1.36 (0.91,2.02)	0.133	0.906
PFTrDA (ng/mL)					
Common cold	1.10 (0.74,1.65)	0.627	0.98 (0.62,1.55)	0.926	0.802
Bronchitis	1.19 (0.83,1.70)	0.350	1.10 (0.73,1.64)	0.658	0.665
Pneumonia	1.20 (0.69,2.11)	0.517	0.94 (0.53,1.67)	0.834	0.612
Tonsillitis	1.21 (0.80,1.84)	0.374	1.46 (0.89,2.40)	0.137	0.460
PFHxS (ng/mL)					
Common cold	0.83 (0.55,1.24)	0.362	1.46 (0.91,2.35)	0.116	0.066
Bronchitis	0.91 (0.64,1.29)	0.599	1.21 (0.84,1.75)	0.301	0.264
Pneumonia	1.29 (0.78,2.14)	0.324	0.95 (0.58,1.56)	0.850	0.320
Tonsillitis	1.09 (0.72,1.65)	0.691	1.05 (0.69,1.60)	0.805	0.766
PFOS (ng/mL)					
Common cold	0.87 (0.66,1.15)	0.341	1.08 (0.78,1.51)	0.635	0.287
Bronchitis	0.96 (0.76,1.22)	0.747	1.02 (0.77,1.36)	0.898	0.823
Pneumonia	1.00 (0.70,1.43)	1.000	1.05 (0.72,1.55)	0.787	0.911
Tonsillitis	1.04 (0.78,1.38)	0.810	0.90 (0.63,1.28)	0.560	0.837

	Boys		Girls		$P_{interaction}$
	OR (95% CI)	*P*	OR (95% CI)	*P*	
6:2 Cl-PFESA (ng/mL)					
Common cold	1.04 (0.75,1.43)	0.818	0.94 (0.66,1.34)	0.733	0.778
Bronchitis	1.11 (0.85,1.45)	0.462	1.01 (0.73,1.41)	0.946	0.606
Pneumonia	1.03 (0.70,1.53)	0.877	0.98 (0.61,1.58)	0.930	0.656
Tonsillitis	1.15 (0.84,1.59)	0.390	1.19 (0.82,1.72)	0.369	0.612
8:2 Cl-PFESA (ng/mL)					
Common cold	1.04 (0.77,1.42)	0.778	0.87(0.65, 1.18)	0.376	0.467
Bronchitis	1.01 (0.78,1.30)	0.939	1.14(0.87, 1.49)	0.338	0.657
Pneumonia	1.10 (0.76,1.60)	0.610	0.94 (0.64, 1.39)	0.775	0.465
Tonsillitis	1.10 (0.81,1.49)	0.539	1.11 (0.81, 1.52)	0.526	0.818

Abbreviation: OR, odds ratio; CI, confidence interval; $P_{interaction}$, the interaction between PFASs and sex. The figure shows the results of Logistic regression after correction for covariates. The model was adjusted for maternal age (continuous variable), family annual income (< 100,000 yuan; ≥ 100,000 yuan), child sex (male; female), gestational week (continuous variable), parity (primipara; multipara), breastfeeding duration (<6 months; ≥6 months), and child exposure to passive smoking (yes; no). These potential confounders were adjusted for in the regression model.

Table S5. Association between single PFASs and the frequency of RTIs in the study population stratified by sex.

	Boys		Girls		*P* interaction
	OR (95% CI)	*P*	OR (95% CI)	*P*	
PFOA (ng/mL)					
Common cold	-0.15 (-0.30,0.01)	0.060	0.12 (-0.05,0.29)	0.170	0.054
Bronchitis	0.02 (-0.23,0.26)	0.900	0.18 (-0.13,0.48)	0.256	0.571
Pneumonia	-0.14 (-0.73,0.45)	0.647	-0.58 (-1.26,0.09)	0.091	0.188
Tonsillitis	-0.20 (-0.55,0.16)	0.274	0.29 (-0.13,0.71)	0.182	0.057
PFNA (ng/mL)					
Common cold	-0.08 (-0.21,0.05)	0.238	0.02 (-0.12,0.16)	0.781	0.451
Bronchitis	0.00 (-0.22,0.22)	0.980	0.02 (-0.25,0.30)	0.864	0.74
Pneumonia	-0.16 (-0.61,0.30)	0.505	-0.29 (-0.91,0.32)	0.353	0.404
Tonsillitis	0.14 (-0.15,0.44)	0.341	0.34 (0.00,0.69)	0.051	0.121
PFDA (ng/mL)					
Common cold	-0.02 (-0.13,0.09)	0.729	0.09 (-0.03,0.21)	0.152	0.327
Bronchitis	-0.01 (-0.19,0.17)	0.884	0.03 (-0.19,0.24)	0.796	0.706
Pneumonia	-0.10 (-0.51,0.30)	0.626	-0.30 (-0.80,0.21)	0.248	0.252
Tonsillitis	0.16 (-0.09,0.40)	0.216	**0.32 (0.03,0.62)**	**0.028***	0.246
PFUnDA (ng/mL)					
Common cold	0.02 (-0.10,0.14)	0.726	0.11 (-0.02,0.24)	0.085	0.516
Bronchitis	0.03 (-0.17,0.22)	0.802	0.02 (-0.21,0.25)	0.878	0.539
Pneumonia	-0.06 (-0.49,0.36)	0.776	-0.14 (-0.67,0.40)	0.621	0.497
Tonsillitis	0.08 (-0.19,0.35)	0.584	**0.41 (0.10,0.73)**	**0.010***	0.068
PFDoDA (ng/mL)					
Common cold	0.04 (-0.06,0.14)	0.390	0.09 (-0.01,0.19)	0.069	0.757
Bronchitis	0.07 (-0.09,0.24)	0.395	-0.03 (-0.20,0.14)	0.743	0.244
Pneumonia	0.09 (-0.31,0.49)	0.664	-0.07 (-0.46,0.32)	0.714	0.39
Tonsillitis	**0.25 (0.02,0.48)**	**0.034***	**0.34 (0.09,0.60)**	**0.008***	0.422
PFTrDA (ng/mL)					
Common cold	0.08 (-0.05,0.21)	0.211	0.09 (-0.03,0.22)	0.147	0.978
Bronchitis	0.15 (-0.07,0.36)	0.179	-0.06 (-0.28,0.17)	0.634	0.161
Pneumonia	0.05 (-0.43,0.53)	0.842	-0.09 (-0.60,0.41)	0.721	0.673
Tonsillitis	0.21 (-0.09,0.50)	0.17	0.23 (-0.09,0.54)	0.161	0.595
PFHxS (ng/mL)					
Common cold	-0.06 (-0.18,0.06)	0.316	-0.01 (-0.12,0.09)	0.790	0.644
Bronchitis	-0.08 (-0.27,0.11)	0.411	-0.03 (-0.23,0.18)	0.811	0.845
Pneumonia	0.26 (-0.15,0.68)	0.216	0.06 (-0.33,0.45)	0.763	0.268
Tonsillitis	0.07 (-0.20,0.34)	0.633	-0.15(-0.44,0.15)	0.327	0.718
PFOS (ng/mL)					
Common cold	-0.02 (-0.1,0.06)	0.62	0.00 (-0.09,0.08)	0.928	0.885
Bronchitis	-0.06 (-0.19,0.08)	0.41	-0.08 (-0.25,0.08)	0.336	0.497
Pneumonia	-0.04 (-0.36,0.27)	0.795	-0.03 (-0.38,0.32)	0.852	0.701
Tonsillitis	0.08 (-0.1,0.26)	0.365	-0.19 (-0.43,0.05)	0.116	0.135

	Boys		Girls		$P_{\text{interaction}}$
	OR (95% CI)	*P*	OR (95% CI)	*P*	
6:2 Cl-PFESA (ng/mL)					
Common cold	0.02 (-0.08,0.11)	0.737	-0.01 (-0.11,0.09)	0.836	0.501
Bronchitis	0.10 (-0.06,0.25)	0.214	0.01 (-0.18,0.19)	0.924	0.192
Pneumonia	-0.11 (-0.44,0.23)	0.531	-0.19 (-0.62,0.25)	0.404	0.328
Tonsillitis	0.09 (-0.12,0.31)	0.404	0.14 (-0.08,0.37)	0.212	0.268
8:2 Cl-PFESA (ng/mL)					
Common cold	0.02 (-0.07,0.10)	0.692	0.00 (-0.09,0.08)	0.911	0.562
Bronchitis	-0.01 (-0.15,0.13)	0.918	0.05 (-0.10,0.20)	0.495	0.942
Pneumonia	-0.14 (-0.47,0.20)	0.422	-0.12 (-0.47,0.23)	0.505	0.744
Tonsillitis	0.10 (-0.09,0.29)	0.300	0.16 (-0.03,0.35)	0.103	0.482

Abbreviation: OR, odds ratio; CI, confidence interval; $P_{\text{interaction}}$, the interaction between PFASs and sex. The figure shows the results of Poisson regression after correction for covariates. The model was adjusted for maternal age (continuous variable), family annual income (<100,000 yuan; ≥100,000 yuan), child sex (male; female), gestational week (continuous variable), parity (primipara; multipara), breastfeeding duration (<6 months; ≥6 months), and child exposure to passive smoking (yes; no). These potential confounders were adjusted for in the regression model. $P_{\text{interaction}}$: the interaction between PFASs and sex. *: P <0.05.

Table S6. Association between PFASs mixture and the incidence of RTIs based on Quantile g-computation analysis.

	OR (95% CI)	*P*	The sum of the positive association	The sum of the negative association
Common cold	0.03 (-0.26,0.31)	0.860	0.411	-0.386
Bronchitis	0.00 (-0.24,0.24)	0.987	0.603	-0.601
Pneumonia	0.14 (-0.21,0.49)	0.430	0.518	-0.378
Tonsillitis	0.10 (-0.19,0.39)	0.493	0.792	-0.691

Abbreviation: OR, odds ratio; CI, confidence interval. The model was adjusted for maternal age (continuous variable), family annual income (<100,000 yuan; ≥100,000 yuan), child sex (male; female), gestational week (continuous variable), parity (primipara; multipara), breastfeeding duration (<6 months; ≥6 months), and child exposure to passive smoking (yes; no). These potential confounders were adjusted for in the regression model.

Table S7. Association between PFASs mixture and the frequency of RTIs based on Quantile g-computation analysis.

	RR (95% CI)	*P*	The sum of the positive association	The sum of the negative association
Common cold	0.00 (-0.07,0.08)	0.933	0.216	-0.212
Bronchitis	-0.06 (0.20,0.07)	0.331	0.358	-0.425
Pneumonia	-0.02 (0.33,0.29)	0.916	0.667	-0.683
Tonsillitis	0.10 (-0.08,0.29)	0.285	1.100	-1.000

Abbreviation: RR, relative risk; CI, confidence interval. The model was adjusted for maternal age (continuous variable), family annual income (<100,000 yuan; ≥100,000 yuan), child sex (male; female), gestational week (continuous variable), parity (primipara; multipara), breastfeeding duration (<6 months; ≥6 months), and child exposure to passive smoking (yes; no). These potential confounders were adjusted for in the regression model.

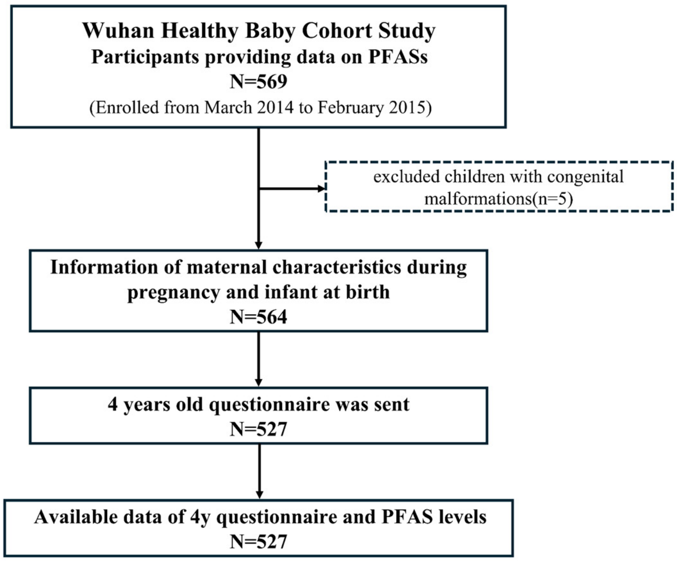

Figure S1. Number of observations at different stages of study.

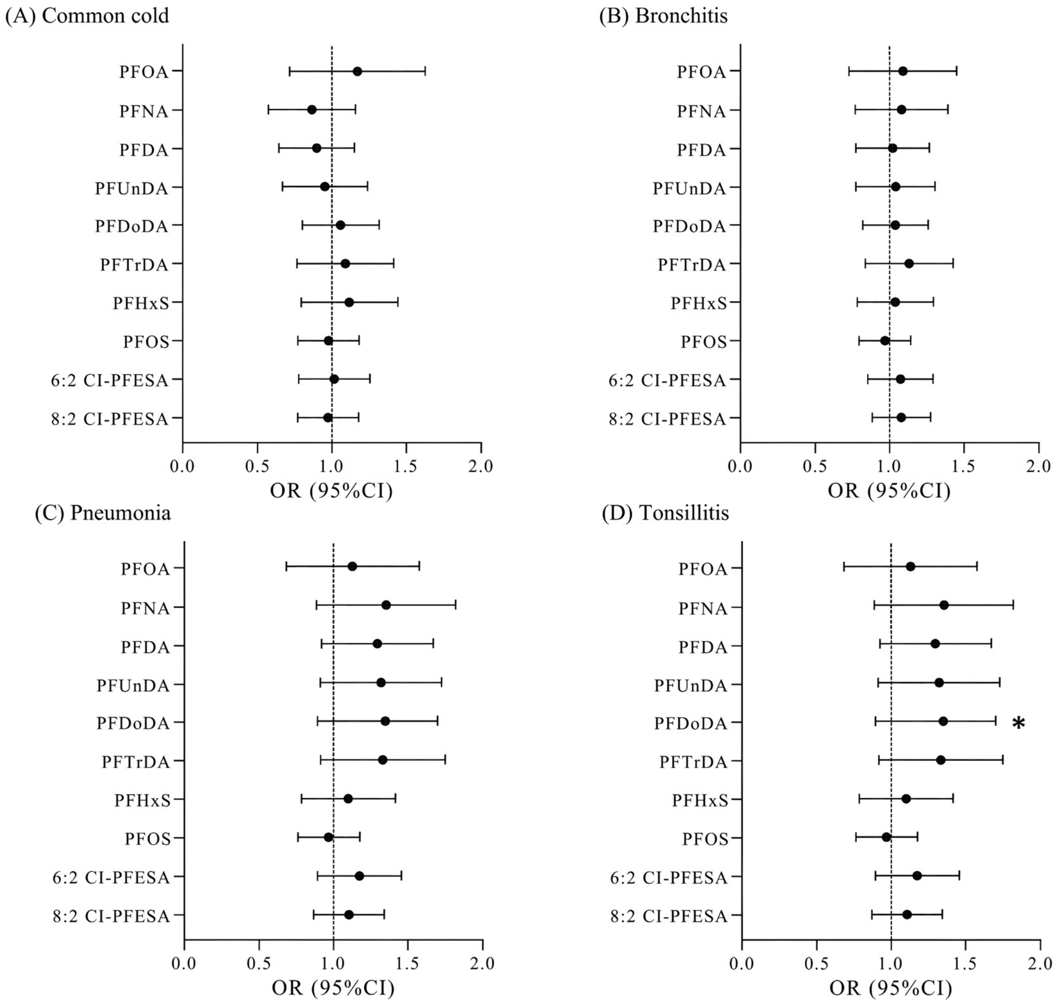

Figure S2. Association between single PFASs and the incidence of RTIs (PFASs as continuous variables). Abbreviation: OR, odds ratio; CI, confidence interval. The figure shows the results of Logistic regression after correction for covariates. The model was adjusted for maternal age (continuous variable), family annual income (<100,000 yuan; ≥100,000 yuan), child sex (male; female), gestational week (continuous variable), parity (primipara; multipara), breastfeeding duration (<6 months; ≥6 months), and child exposure to passive smoking (yes; no). These potential confounders were adjusted for in the regression model. *: p<0.05

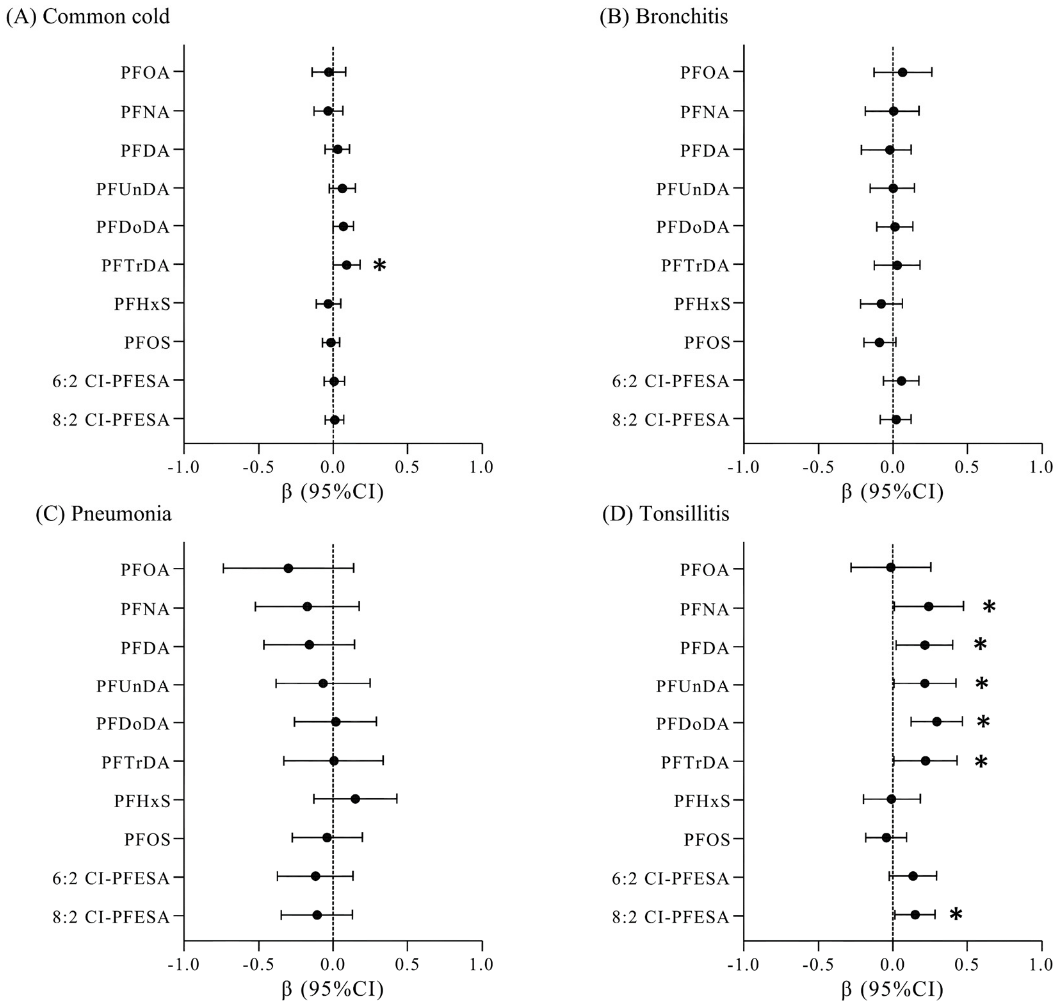

Figure S3. Association between single PFASs and the frequency of RTIs (PFASs as continuous variables). Abbreviation: CI, confidence interval. The figure shows the results of Poisson regression after correction for covariates. The model was adjusted for maternal age (continuous variable), family annual income (<100,000 yuan; ≥100,000 yuan), child sex (male; female), gestational week (continuous variable), parity (primipara; multipara), breastfeeding duration (<6 months; ≥6 months), and child exposure to passive smoking (yes; no). These potential confounders were adjusted for in the regression model. *: $p<0.05$

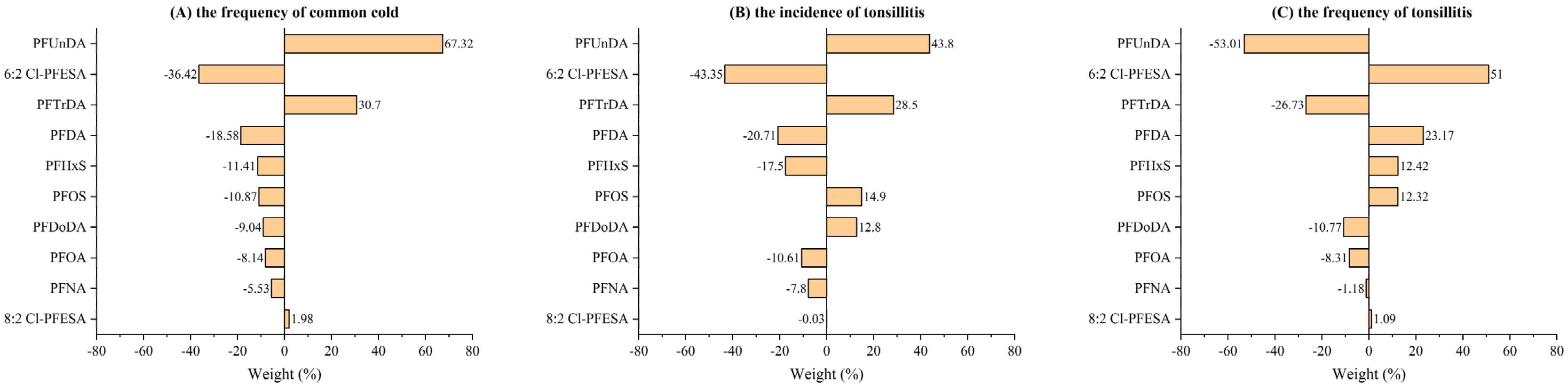

Figure S4. Weights of each single PFASs contributing to the association between PFASs mixture and RTIs. The figure shows the results of Quantile g-computation analysis.
